# Elemental Fingerprinting Combined with Machine Learning Techniques as a Powerful Tool for Geographical Discrimination of Honeys from Nearby Regions

**DOI:** 10.3390/foods13020243

**Published:** 2024-01-12

**Authors:** Andrea Mara, Matteo Migliorini, Marco Ciulu, Roberto Chignola, Carla Egido, Oscar Núñez, Sònia Sentellas, Javier Saurina, Marco Caredda, Mario A. Deroma, Sara Deidda, Ilaria Langasco, Maria I. Pilo, Nadia Spano, Gavino Sanna

**Affiliations:** 1 Department of Chemical, Physical, Mathematical and Natural Sciences, University of Sassari, Via Vienna 2, 07100 Sassari, Italy; a.mara@studenti.uniss.it (A.M.); saradeidda96@tiscali.it (S.D.); ilangasco@uniss.it (I.L.); mpilo@uniss.it (M.I.P.); nspano@uniss.it (N.S.); 2 Department of Biotechnology, University of Verona, Strada le Grazie 15, 37134 Verona, Italy; matteo.migliorini@univr.it (M.M.); marco.ciulu@univr.it (M.C.); roberto.chignola@univr.it (R.C.); 3 Department of Chemical Engineering and Analytical Chemistry, University of Barcelona, Martí i Franquès 1-11, 08028 Barcelona, Spain; cegido.perianes@gmail.com (C.E.); oscar.nunez@ub.edu (O.N.); sonia.sentellas@ub.edu (S.S.); xavi.saurina@ub.edu (J.S.); 4 Research Institute in Food Nutrition and Food Safety, University of Barcelona, Recinte Torribera, Av. Prat de la Riba 171, Edifici de Recerca (Gaudí), Santa Coloma de Gramenet, 08921 Barcelona, Spain; 5 Serra Húnter Fellow, Departament de Recerca i Universitats, Generalitat de Catalunya, Via Laietana 2, 08003 Barcelona, Spain; 6 Department of Animal Science, AGRIS Sardegna, Loc. Bonassai, 07100 Sassari, Italy; mcaredda@agrisricerca.it; 7 Department of Agriculture, University of Sassari, Viale Italia, 39A, 07100 Sassari, Italy; mderoma@uniss.it

**Keywords:** honey, geographical classification, botanical classification, elements, ICP-MS

## Abstract

Discrimination of honey based on geographical origin is a common fraudulent practice and is one of the most investigated topics in honey authentication. This research aims to discriminate honeys according to their geographical origin by combining elemental fingerprinting with machine-learning techniques. In particular, the main objective of this study is to distinguish the origin of unifloral and multifloral honeys produced in neighboring regions, such as Sardinia (Italy) and Spain. The elemental compositions of 247 honeys were determined using Inductively Coupled Plasma Mass Spectrometry (ICP-MS). The origins of honey were differentiated using Principal Component Analysis (PCA), Linear Discriminant Analysis (LDA), and Random Forest (RF). Compared to LDA, RF demonstrated greater stability and better classification performance. The best classification was based on geographical origin, achieving 90% accuracy using Na, Mg, Mn, Sr, Zn, Ce, Nd, Eu, and Tb as predictors.

## 1. Introduction

Honey is a natural sweet food produced by bees (*Apis mellifera*) with numerous nutraceutical and therapeutical properties [1,2,3,4,5,6,7]. Several factors, including botanical origin, environmental conditions, and bee species, impact the composition of honey and play a key role in determining its sensorial and health-promoting properties [7].

Owing to its unique qualities, honey is particularly vulnerable to unauthorized food manipulation. Adulteration and mislabeling are the most prevalent forms of honey fraud [8]. According to European (EU) legislation [9], honey labeling must indicate EU or non-EU origins, whereas botanical and local geographical origins are optional [10]. Nevertheless, because these factors strongly affect the economic value and price of honey, they are frequently falsified. This type of counterfeiting has a remarkable economic impact, especially on small beekeepers who tend to produce rare or unifloral honey [11]. For these reasons, there is considerable scientific interest in developing innovative analytical methods to verify the honey authenticity [12].

Honey’s botanical origin is generally ascertained through melissopalynological analysis. However, this method is ineffective if the honey has been filtered. On the other hand, there are no standard methods for determining geographical origin. Current methods involve advanced analytical tools coupled with chemometrics [13]. Physicochemical [14,15,16,17], elemental [18], isotopic [19], chromatographic, and hyphenated mass spectrometry methods [20,21], NMR [22,23], DNA-based [24,25], electronic sensing [26,27], and spectroscopic [28,29,30,31,32] techniques are nowadays the most used for predicting botanical and geographical origin, or detecting adulterants [33]. Usually, botanical issues are investigated using chromatographic techniques and physicochemical analyses [12,14,34]. However, while vibrational spectroscopy is commonly used to detect adulterated honeys [12,33], it is also highly effective in predicting the authenticity of almost all types of honey. Furthermore, the use of portable instrumentation enhances the versatility of this approach [35,36]. Finally, elemental and isotopic methods are prevalently used for geographical origin discrimination [19,37], but they are also useful in determining botanical origin [37,38]. Elements in honey reflect the soil composition [39], flora [40], and anthropogenic activities [41]. The translocation of elements is influenced by season [42], bee species [43], and, in general, by environmental conditions [44]. Furthermore, honey reflects the urbanization grade [39,45,46]. All these factors determine the elemental fingerprint of honey, allowing its authentication in terms of both geographical and botanical origins [47].

The elemental composition of honey has been characterized worldwide. Recent studies investigated honeys from Argentina [48], Bulgaria [40,49,50], China [43,51,52,53], Croatia [42,49,54,55], Denmark [56], Egypt [57], Eritrea [58], Ethiopia [59], France [60], Germany [56], Greece [37,57,61], Hungary [62,63], Israel [64], Italy [37,38,46,65,66,67,68,69,70,71,72,73], Kazakhstan [73], Kosovo [74], Montenegro [75], Morocco [49,57], New Zealand [45,56], Poland [37], Romania [37,60], Serbia [74], Slovenia [49], Spain [57,76,77,78,79], Thailand [39], Tunisia [68], and Turkey [16,49]. Typically, data management and elaboration involve multivariate analysis and machine-learning techniques, including Principal Component Analysis (PCA) [38,59], Cluster Analysis (CA) [37,39], Linear Discriminant Analysis (LDA) [57,59], Partial Least Squares regression (PLS) [37,52], Decision Tree Analysis (DTA) [56], Self-Organizing Maps (SOMs) [40], and Soft Independent Modelling of Class Analogy (SIMCA) [60]. Few papers in the literature report cases where elemental fingerprinting has discriminated honeys according to their botanical and geographical origin [37,42,49,60]. Research often focuses on authenticating honeys from various origins based on botanical sources or regions with unique climates, soils, or levels of urbanization. European legislation requires differentiation between EU and non-EU honeys, which is usually feasible due to the numerous environmental factors that affect their elemental fingerprints. On the other hand, discriminating honey from nearby areas is a more challenging task.

The main goal of this study is to distinguish unifloral and multifloral honeys from two nearby regions. In this context, Sardinia (Italy) and Spain have been chosen as case studies. Geologically, the Corsica–Sardinian microplate separated from the Iberian Peninsula during the Miocene. Therefore, the soil composition of Sardinia is more similar to that of southeastern Spain than Italy [80]. In addition, the two regions have similar climatic conditions.

A comprehensive selection of multifloral and unifloral honeys from common botanical species, such as rosemary and eucalyptus, was considered for this purpose. The physicochemical characterizations of eucalyptus and rosemary honeys from Spain [81,82] and Italy [83,84] have been reported in literature. Possible differences among the eucalyptus and rosemary honeys are related to their diastase activity and acidity parameters. The diastase activity of Italian eucalyptus honey samples is higher than that of Spanish ones, whereas the acidity parameters of Spanish eucalyptus honey samples are higher than those of Italian ones. Additionally, Spanish rosemary honey samples exhibit higher diastase activity than Italian ones. Furthermore, some typical unifloral honeys from Sardinia, such as strawberry tree, asphodel, and thistle honey [85], were analyzed. Strawberry-tree honey is famous worldwide for its unique “bitter” taste [86] and for its healing properties [87]. The chemical composition of strawberry-tree honey confirms its botanical origin due to the presence of high concentrations (several hundred mg kg^−1^) of homogentisic acid [86,88], a compound not found in other unifloral honeys. The botanical origin of asphodel honey is also guaranteed by the peculiar presence of methyl syringate in it [89]. Among all the Sardinian unifloral honeys considered in this study, the thistle honey is characterized by the lowest values of pH, electrical conductivity, and color [14]. The honeys’ elemental fingerprints were determined using a validated Inductively Coupled Plasma Mass Spectrometry (ICP-MS) method [38]. Four major elements (Na, Mg, K, Ca), twenty-three among trace and toxic elements (Ag, As, Ba, Be, Bi, Cd, Co, Cr, Cu, Fe, Hg, Li, Mn, Mo, Ni, Pb, Sb, Sn, Sr, Te, Tl, V, Zn) and fourteen lanthanides (La, Ce, Pr, Nd, Sm, Eu, Gd, Tb, Dy, Ho, Er, Tm, Yb, Lu) were analyzed. The data were processed using PCA for multivariate data visualization, whereas LDA and Random Forest were used for honey classification.

## 2. Materials and Methods

### 2.1. Honey Samples

**Figure 1:**
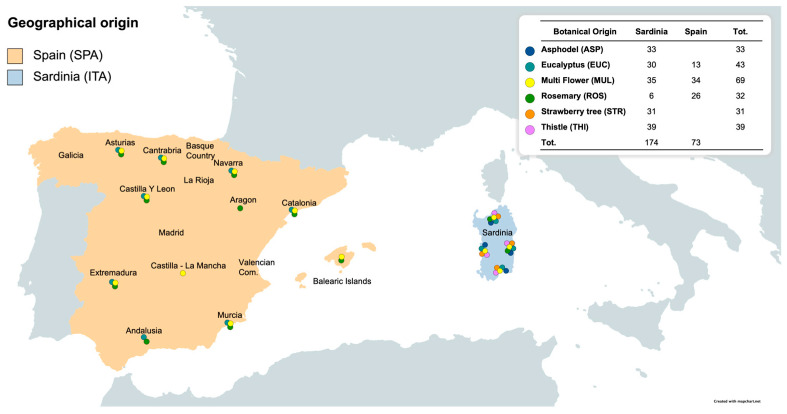
Geographical and botanical origins of honey samples.

The study analyzed honeys from two areas in the Western Mediterranean: Spain (SPA) and Sardinia (Italy, ITA). The geographical and botanical origins of the honey are shown in Figure 1.

In total, 247 honey samples from Spain (SPA = 73) and Sardinia (ITA = 174) were examined. The sample set consisted of both multifloral and unifloral honeys. Among the common honeys from the two regions, multifloral (MUL, SPA = 34, ITA = 35), eucalyptus (EUC, SPA = 13, ITA = 30), and rosemary (ROS, SPA = 26, ITA = 6) were included. Additionally, three characteristic unifloral Sardinian honeys, asphodel (ASP, ITA = 33), strawberry tree (STR, ITA = 31), and thistle (THI, ITA = 39), were considered for the geographical attribution. The botanical origin of the samples was determined by melissopalynological analysis. The collection was recorded between 2020 and 2022, reflecting the flowering and seasonality of the botanical sources. In general, eucalyptus, thistle, and multifloral honeys were produced in spring, summer, and fall. Rosemary and strawberry-tree honeys were collected in fall and winter, while asphodel honeys were produced in winter and spring. Sardinian honeys were gathered throughout the island (Figure 1), whereas the Spanish honeys were from Andalusia, Aragon, Asturias, Cantabria, Castilla la Mancha, Castilla-Leon, Catalonia, Extremadura, Balearic Islands, Navarre, and Basque Country (Figure 1). Previous studies [81] have reported details on the chemical–physical and sensory characteristics of the honeys, such as color, pH, moisture, taste, and botanical markers. Honeys were stored in the dark at 4 °C until analysis.

### 2.2. Instrumentation and Reagents

The elemental analysis was performed using a NexION 300X ICP-MS spectrometer from Perkin Elmer (Milan, Italy). The spectrometer was equipped with an S10 autosampler, glass concentric nebulizer, glass cyclonic spray chamber, and kinetic energy discrimination (KED) collision cell. Microwave acid digestion was performed using an ultraWAVE™ from Milestone (Sorisole, Italy) equipped with a single reaction chamber (SCR) system, a fifteen-position rotor, and polytetrafluoroethylene (PTFE) vessels (15 cm^3^). Dry ashing was performed using a Controller P320 muffle from Nabertherm (Lilienthal, Germany). Samples were homogenized before analysis using an Ultraturrax mixer model T18 (Staufen, Germany). Nylon filters (pore diameter, 0.22 μm), syringes, metal-free polypropylene tubes (15 and 50 cm^3^), and porcelain crucibles (150 cm^3^) were supplied by VWR (Milan, Italy).

A MilliQ Plus System (Millipore, Vimodrone, Italy) was used to produce type I water (resistivity > 18 MΩ cm^−1^). Nitric acid (67–69% *w*/*w*, NORMATON^®^ for ultra-trace analysis) and hydrogen peroxide (30% *w*/*w*, NORMATON^®^ for ultra-trace metal analysis) were supplied by VWR (Milan, Italy). Periodic table mix 1 for ICP (TraceCert^®^, 33 elements, 10 mg dm^−3^ in 10% HNO_3_), periodic table mix 3 for ICP (TraceCert^®^, 16 elements, 10 mg dm^−3^ in 5% HNO_3_), Rh solution (1000 mg dm^−3^ in 3% HNO_3_), apple leaves NIST SRM^®^ 1515 and mussel tissue BCR 668 were from Sigma-Aldrich (St. Louis, MO, USA). Single standard solutions of Na, Mg, K, Ca, Hg, Mo, Sb, and Sn (100–1000 mg dm^−3^ in 2–5% HNO_3_) were obtained from Carlo Erba (Milan, Italy).

### 2.3. Sample Preparation

Sample preparation involved the use of microwave acid digestion and dry ashing techniques. Microwave acid digestion was utilized to determine macroelements, trace elements, and toxic elements, while dry ashing was employed for lanthanide analysis [70]. Microwave acid digestion, performed according to a previously described method [38], was optimized by a 2^2^ full factorial experimental design. In this manner, the residual carbon and acidity levels were minimized, preventing the need for unnecessary dilutions and reducing the matrix effect. Initially, the samples were homogenized at 40 °C. Then, approximately 0.700 g of honey was weighed in 15 cm^3^ PTFE vessels and treated with 0.5 cm^3^ of HNO_3_, 3 cm^3^ of H_2_O_2_, and 4 cm^3^ of type I water. After digestion at 240 °C, samples were collected, diluted to 15 cm^3^, and filtered before analysis. Dry ashing was performed weighing about 5.0 g of honey in porcelain crucibles (150 cm^3^). After ashing at 600 °C, samples were treated with 10 cm^3^ of 5% HNO_3_ aqueous solution, diluted to 15 cm^3^, and filtered before analysis. Table S1 reports the operational conditions of both methods.

### 2.4. Elemental Analysis

A previously developed and validated procedure [38] was used on a NexION 300X ICP-MS (Perkin Elmer) to perform elemental analysis. Here, detailed information regarding instrumental parameters, elemental settings, method assessment, performance, quality control, and validation is reported. The literature method for the analysis of trace and toxic elements (i.e., Ag, As, Ba, Be, Bi, Cd, Co, Cr, Cu, Fe, Hg, Li, Mn, Mo, Ni, Pb, Sb, Sn, Sr, Te, Tl, V, Zn) in honey [38] was implemented to analyze macro elements (i.e., Na, Mg, K, Ca) and lanthanides (i.e., La, Ce, Pr, Nd, Sm, Eu, Gd, Dy, Ho, Er, Tm, Yb, Lu). Tables S2 and S3 report the instrumental conditions and elemental settings for the analysis of macro elements and lanthanides, respectively. Trueness was evaluated by analyzing two certified reference materials, apple leaves NIST SRM^®^ 1515, and mussel tissue BCR 668. The results are presented in Table S4.

### 2.5. Statistical Analysis

Data analysis was conducted using the statistical freeware software R (v. 4.3.1) run in the free integrated development environment R-Studio (v. 2023.3.1), GraphPad Prism (v. 9.1.0 221), and Chemometric Agile Tool (CA) [90]. For data visualization, PCA was performed removing all elements that were rarely quantified. Compositional data analysis (CoDa) was applied as a data pre-treatment before PCA to improve the interpretability using centered log-ratio transformation [91]. To remove missing values (i.e., those below the limit of detection or quantification), the dl23 method (two-thirds of the limit of detection) was used [91]. To classify honeys according to botanical origin, geographical origin, and their combination, LDA was performed. A RF machine-learning algorithm was also used (R package randomForest, [92]). Data for each factor (i.e., geographical, botanical origin, and their combination) were extracted and divided into train and test sets. The train set included an equal number of samples from each group, equivalent to half of the least populated group. The remaining data constituted the test set. The classification algorithms were applied by removing chemical elements with missing (i.e., below detection or quantification limits) or repeated values, iterating 100 times both train and test sampling to increase the statistical significance. Accuracy was measured by averaging over the accuracies obtained in the 100 replicas. In particular, RF analysis was performed using 1000 trees and setting the mtry parameter equal to the square root of the number of predictors [92]. To find the smallest set of chemical elements required to efficiently discriminate between groups, the importance for the classification of variables was first measured using the mean decrease in the Gini index [92,93]. Predictors with the lower Gini index were removed and the analysis was iterated with the remaining predictors. The smallest set of chemical elements was identified as the one preceding a visible reduction in the overall classification accuracy. Statistical significance was set at *p* < 0.05.

## 3. Results

### 3.1. Elemental Fingerprints

Data relative to the elemental composition of honeys from Spain and Sardinia (Italy) are reported in Table S5. For each type of honey, the minimum, average, and maximum concentrations are reported. K was generally the most abundant macroelement in all honeys, followed by Ca, Na, and Mg. The most abundant trace elements were Zn, Cu, Mn, Sr, Ba, Fe, and Ni. The remaining trace elements were present at lower concentrations or below the limit of quantification (LoQ). Li was only quantified in EUC and STR. As, Cd, Pb, Sn, and Tl were rarely quantified, while Be, Bi, Hg, Sb, and Te were always below the relevant LoQs. Finally, the concentrations of lanthanides (range between mg kg^−1^ and ng kg^−1^) followed the pattern predicted by the Oddo-Harkins rule, which holds that the elements with even atomic numbers are more abundant than those with immediately adjacent atomic numbers. Notably, Eu deviated from the expected trend in all Spanish honeys.

### 3.2. Principal Component Analysis

Before conducting the PCA, the data underwent a centered log-ratio transformation. This pre-treatment enhances the interpretability of the PCA outcomes by emphasizing sample percentage compositions. For comparison, Figure S1 shows the PCA performed with standardization. The comparison indicates that the centered log-ratio transformation increases the variance of PC2. Thus, scores and loadings were more scattered.

**Figure 2:**
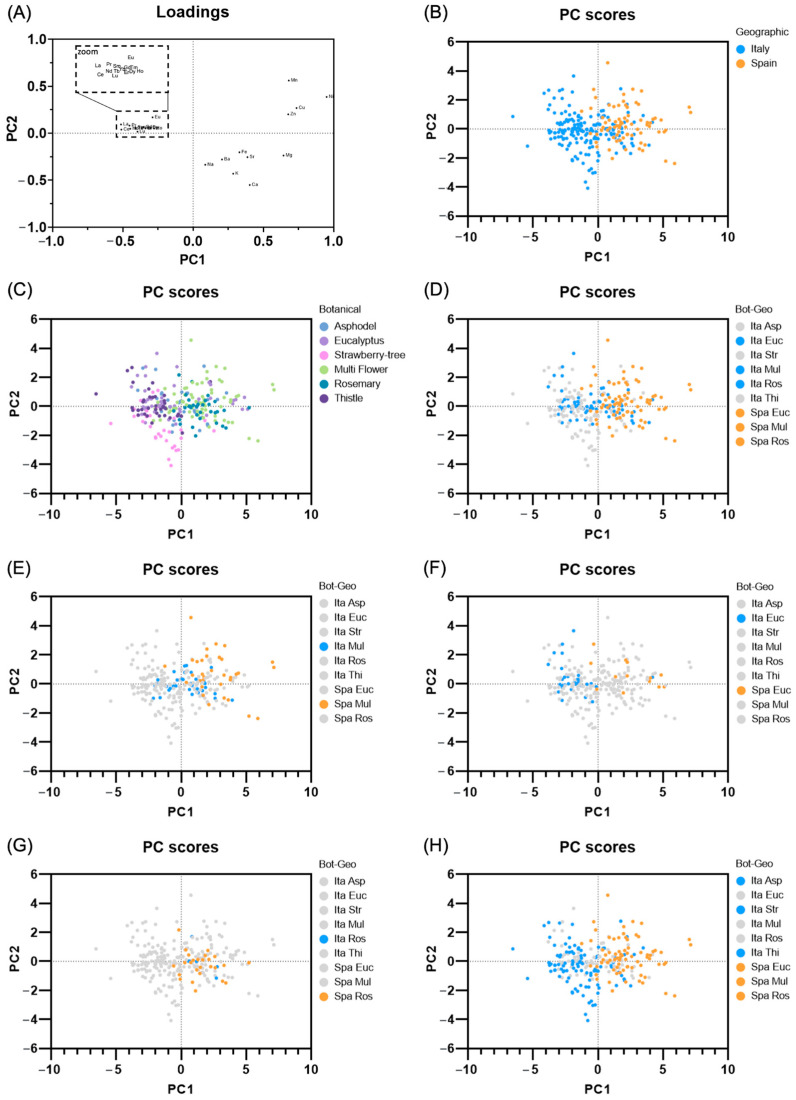
PCA analysis. (**A**) loading plot; (**B**) score plot, object colored according to geographical origin; (**C**) score plot, object colored according to botanical origin; (**D**) score plot, object colored according to common botanical origin; (**E**) score plot, object colored according to multi flower honey; (**F**) score plot, object colored according to eucalyptus honey; (**G**) score plot, object colored according to rosemary honey; (**H**) score plot, object colored according to uncommon origins.

Figure 2 shows the loading and score plots of the PCA, with the first two components accounting for 42.7% and 11.6% of the total variance, respectively (Scree plot, Figure S2).

From the loading plot (Figure 2A), positive PC1 values indicate a greater percentage of macro and trace elements, whereas negative values indicate a higher percentage of lanthanides. On the other hand, PC2 distinguishes by negative values the alkaline and alkaline earth elements (Na, Mg, K, Ca, Ba, Sr), and by positive values the transition metals (Cu, Ni, Mn, Zn). Notably, Fe shows a higher correlation with the cluster formed by alkaline and alkaline-earth elements than with that formed by transition trace elements. Looking at the score plots (Figure 2B–H), objects are colored to highlight honeys according to geographical (Figure 2B) and botanical origins (Figure 2C), common botanical origins to both geographical areas (Figure 2D–G), and uncommon origins (Figure 2H). Overall, samples exhibit differentiation based on geographical origin along PC1 (Figure 2B), whereas the discrimination based on botanical origin is less evident (Figure 2C). However, when considering the unifloral honeys that are common in Sardinia and Spain (Figure 2D–G), the distinction between the two regions is less evident. Only eucalyptus honeys were separated (Figure 2F). Finally, as expected, the groups formed by samples from uncommon origins were the most differentiated ones (Figure 2H).

### 3.3. Classification by LDA and RF

Honeys were classified according to their geographical and botanical origins. For this purpose, LDA and RF were used and compared. Table 1 reports the results obtained.

Both LDA and RF performed well in classifying botanical origin across different categories. Notably, both algorithms accurately predicted the asphodel and strawberry-tree honeys during training and testing. Conversely, multifloral and rosemary honeys were more difficult to classify. In both training and testing sets, these categories show lower accuracy scores than others. The two algorithms demonstrate excellent performance in classifying samples from Sardinia (Italy) and Spain based on their geographical origin. However, when considering both geographical and botanical origins, the accuracy of LDA and RF tends to decrease. Overall, the algorithms were better at predicting geographical origin than botanical origin, while predicting both origins at the same time posed a greater challenge. LDA and RF demonstrated accuracy and competitiveness across various categories and origins. Nevertheless, RF showed more stable performance and better agreement between training and testing.

**Figure 3:**
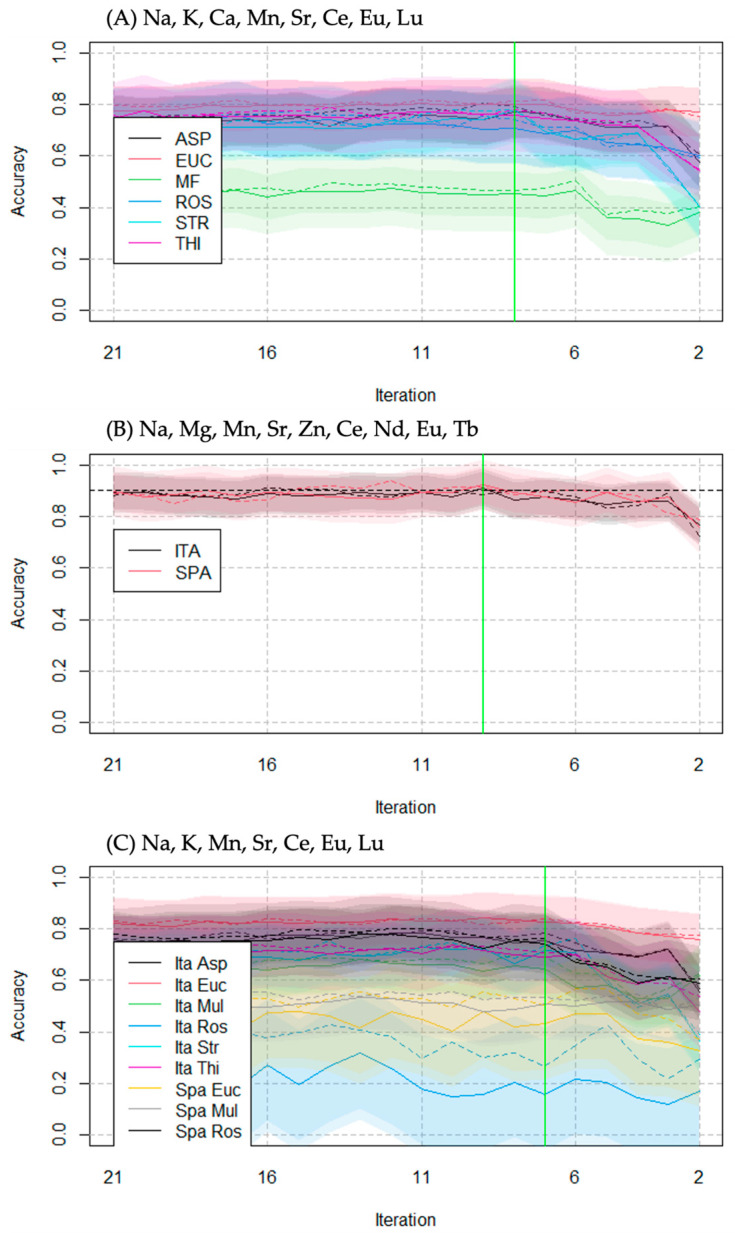
Accuracy of RF algorithm at varying iterations, which indicates the number of predictors (chemical elements) used for classification purposes. Continuous line: accuracy in training. Dashed lines: accuracy in testing. Shades: mean ± standard deviation. Green line: accuracy drop. (**A**) RF classification of honeys based on botanical origin; (**B**) RF classification based on geographical origin; (**C**) RF classification based on both geographical and botanical origin. All values are given in Tables S6–S8.

Additionally, the algorithm allows direct assessment of which elements were the most important for the models. The classification accuracy was evaluated by iterating the calculations while varying the number of predictors (Figure 3). The reported results indicate that Na, K, Ca, Mn, Sr, Ce, Eu, and Lu are the most significant elements for classifying honeys based on their botanical origin. The elements Na, Mg, Mn, Sr, Zn, Ce, Nd, Eu, and Tb are the most effective predictors for classifying geographical origin. Na, K, Mn, Sr, Ce, Eu, and Lu are the most relevant elements for both origin classifications.

## 4. Discussion

Elemental analysis allows the content of toxic and nutritional elements to be evaluated. The levels of harmful elements are similar to or frequently lower than those found in other Spanish [78,79] and Italian [46,69,72] honeys analyzed in previous studies. On the other hand, honeys have a relatively high content of minerals such as Na, Mg, K, Ca, Zn, Mn, and Cu. However, assuming a daily honey intake of 20 g, elements of nutritional interest do not cover daily requirements, while the toxic elements do not pose any health risk.

Elemental fingerprints were tested for classifying honeys according to geographical and botanical origins. PCA was performed using two different data sets pre-treatment, centered log-ratio transformation (Figure 2), and standardization (Figure S1). As previously reported [91], the centered log-ratio transformation improves the data interpretability by distributing a part of the variance explained by the first component into the other components. Consequently, loadings and scores in the first two components are more dispersed (Figure 2), allowing a more comprehensive differentiation of honey categories. The PCA results show that botanical information predominates over geographical information (compare Figure 2B with Figure 2C). Except for rosemary honeys (for which Sardinia is underrepresented), multifloral honeys tend to overlap in the graph (Figure 2E), while eucalyptus honeys are more distinguished (Figure 2D). It is hypothesized that this difference is attributable to the origin of Spanish eucalyptus honeys. These are produced mainly in the northern regions, which are geographically more different from Sardinia. Furthermore, the results of the PCA analysis indicate that geographical discrimination is facilitated, as expected, when considering different botanical origins (compare Figure 2D with Figure 2H). Finally, the score plots also allow the comparison of the honeys’ elemental compositions. Broadly, the percentage of lanthanides is lower in Spanish samples compared with Sardinian unifloral varieties. On the other hand, macroelements and trace elements are relatively less abundant in unifloral honey than in multifloral ones (Figure 2).

Regarding honey authentication, the RF algorithm performs better than LDA in classifying honeys according to their origins. Overall, the classification by geographical origin is the most accurate (90%, predictors: Na, Mg, Mn, Sr, Zn, Ce, Nd, Eu, Tb). The accuracy tends to decrease when classifying honey by botanical origin (73%, predictors: Na, K, Ca, Mn, Sr, Ce, Eu, Lu) and when combining geographical and botanical origins (65%, predictors: Na, K, Mn, Sr, Ce, Eu, Lu). Based on these results, it is possible that geographical origin may have a greater influence than botanical origin on the elemental fingerprint. However, predicting the botanical origin is challenging, and combining the two factors reduces the classification model’s accuracy.

To our knowledge, this is the first case where RF combined with elemental fingerprinting has been used to distinguish the origin of honey. Thus, the accuracy of the models cannot be easily assessed if compared with data presented in the literature. Few studies have investigated the authentication of honey in terms of both geographical and botanical origin [37,42,49,60]. Often the data were not processed with classification algorithms [42,49]. Drivelos et al. achieved excellent results in geographical classification [37]. Magdas et al. also achieved excellent results, but their classification models were obtained using isotopic markers in addition to elements [60].

In general, as expected, the models obtained indicate that unifloral varieties are easier to accurately classify than multifloral varieties of honey. Regarding elemental predictors, Na, Mn, Sr, Ce, and Eu are common to all origin classifications, while Mg, Zn, Nd, and Tb are useful for geographical classification. On the other hand, K, Ca, and Lu are relevant for botanical origin. These findings can be compared and align with those of Pavlin et al. [49], who analyzed different varieties of unifloral and multifloral honeys from Slovenia, Croatia, Bulgaria, Turkey, and Morocco [49]. They reported Mn, K, and Ca as botanical markers, and Na, Mg, and Fe as geographical ones. However, the labeling of elements as markers for the determination of a specific origin may vary depending on the honey’s origin or variety.

Regarding lanthanides, they have primarily been reported as indicators of geographical origin [19,60]. In this study, Ce, Nd, Eu, and Tb are significant for geographical classification, while Ce, Eu, and Lu are significant for botanical classification. Previously, Squadrone et al. [66] reported that lanthanides can help in discriminating unifloral and multifloral honey using the ratio of light and heavy rare earths (LREE/HREE), while Gulino et al. [70] reported that the fractionation of heavy lanthanides partially helps in geographical classification. The results of this research suggest that Ce and Eu are among the most important lanthanides in all models. Eu exhibits anomalous behavior in Spanish honeys, which is not observed in those from Sardinia. Gulino et al. [70] suggested that these anomalies could be attributed to Ba interferences during the analyses. Although it may be possible, any potential bias would affect all samples and should be highly correlated with Ba. However, the level of correlation between these parameters is low (r = 0.16), so this hypothesis can be ruled out. Based on the results obtained, Eu may be considered a reliable marker in all classification models.

## 5. Conclusions

In conclusion, the analysis of macroelements, trace elements, toxic elements, and lanthanides allows for the assessment of their potential use as markers for the botanical and geographical classification of honeys. Specifically, the investigation compared honey originating from the same botanical source but produced in neighboring geographic locations for climate, flora, and geology. The accuracy of honey classification based on geography is reliable when comparing honey of different botanical origins but tends to decrease when comparing the same botanical varieties. As expected, multifloral honeys are more difficult to classify in terms of both botanical and geographical origin.

This study confirmed the usefulness of elemental fingerprinting and suggested its potential use to effectively discriminate honeys from similar regions. One possible application could be to discriminate European honeys from those produced in neighboring non-EU countries, or those that share similar Mediterranean floral resources. In future, honeys from these regions will be studied.

## Figures and Tables

**Table 1:** Linear Discrimination Analysis (LDA) and Random Forest (RF) for honey classification according to their origins. Results are expressed as percentage accuracy (±standard deviation).

Geographical Origin	LDA		Random Forest	
	Train	Test	Train	Test
ITA	91 ± 5	78 ± 6	91 ± 3	88 ± 4
SPA	92 ± 4	79 ± 8	92 ± 3	91 ± 5
**Botanical Origin**	**LDA**		**Random Forest**	
	**Train**	**Test**	**Train**	**Train**
ASP	91 ± 6	83 ± 9	77 ± 9	79 ± 9
EUC	84 ± 8	63 ± 9	80 ± 10	81 ± 9
MUL	80 ± 10	50 ± 10	45 ± 10	47 ± 7
ROS	74 ± 9	50 ± 15	70 ± 10	70 ± 10
STR	90 ± 5	70 ± 10	78 ± 8	80 ± 10
THI	76 ± 9	60 ± 10	76 ± 9	78 ± 9
**Geographical and Botanical ** **Origins**	**LDA**		**Random Forest**	
	**Train**	**Test**	**Train**	**Test**
ITA ASP	90 ± 7	82 ± 9	75 ± 10	78 ± 9
ITA EUC	89 ± 6	80 ± 10	83 ± 7	84 ± 9
ITA MUL	80 ± 7	60 ± 10	65 ± 10	69 ± 9
ITA ROS	98 ± 7	50 ± 30	20 ± 30	30 ± 20
ITA STR	89 ± 6	70 ± 10	70 ± 10	70 ± 10
ITA THI	74 ± 9	60 ± 10	69 ± 8	70 ± 10
SPA EUC	80 ± 15	40 ± 20	40 ± 30	50 ± 20
SPA MUL	70 ± 10	30 ± 10	50 ± 10	50 ± 10
SPA ROS	75 ± 10	50 ± 20	74 ± 9	80 ± 10

## Data Availability

Data is contained within the article or supplementary material.

## Supplementary Materials

The following supporting information can be downloaded at: https://www.mdpi.com/article/10.3390/foods13020243/s1, Figure S1: Principal Component Analysis (PCA) performed using autoscaling as data pre-treatment. (A) Loading plot; (B) Score plot; Figure S2: Proportion of variance (PCA using centered log-ratio transformation); Table S1: Operational conditions of microwave acid digestion and dry ashing for honey elemental analysis; Table S2: Inductively Coupled Plasma–Mass Spectrometry (ICP-MS) instrumental conditions for honey elemental analysis; Table S3: Elemental settings and validation parameters for the ICP-MS determination of macroelements and lanthanides in honey; Table S4: Trueness evaluation for the ICP-MS determination of macroelements and lanthanides by means of analysis of certified reference materials (NIST SRM 1515 apple leaves and BCR 668 mussel tissue); Table S5: Concentration of macroelements, trace elements, toxic elements and lanthanides in honeys from Sardinia and Spain (min; mean ± sd; max); Table S6: Random forest (RF) accuracy table for each set of predictors tested: botanical origin; Table S7: RF accuracy table for each set of predictors tested: geographical and botanical origin; Table S8: RF accuracy table for each set of predictors tested: geographical origin.
