# Supplementary material for: Elemental Fingerprinting Combined with Machine Learning Techniques as a Powerful Tool for Geographical Discrimination of Honeys from Nearby Regions
Source: Foods. 2024 Jan 12;13(2):243. doi: 10.3390/foods13020243 (PMC10814624; doi:10.3390/foods13020243)

Elemental fingerprinting combined with machine learning techniques as a powerful tool for geographical discrimination of honeys from nearby regions

Andrea Mara[1], Matteo Migliorini[2], Marco Ciulu[2], Roberto Chignola[2], Carla Egido[3], Oscar Nunez[3,4,5], Sònia Sentellas[3,4,5], Javier Saurina[3,4], Marco Caredda[6], Mario A. Deroma[7], Sara Deidda[1], Ilaria Langasco[1], Maria I. Pilo[1], Nadia Spano[1], and Gavino Sanna[1].

[1] Department of Chemical, Physical, Mathematical and Natural Sciences. University of Sassari. Via Vienna 2, 07100 Sassari, Italy.

[2] Department of Biotechnology, Food Analysis Lab, University of Verona. Strada le Grazie 15, 37134 Verona, Italy.

[3] Department of Chemical Engineering and Analytical Chemistry, University of Barcelona. Martí i Franquès 1-11, 08028 Barcelona, Spain.

[4] Research Institute in Food Nutrition and Food Safety, University of Barcelona, Recinte Torribera, Av. Prat de la Riba 171, Edifici de Recerca (Gaudí), Santa Coloma de Gramenet, E08921 Barcelona, Spain.

[5] Serra Húnter Fellow, Departament de Recerca I Universitats, Generalitat de Catalunya, Via Laietana 2, E08003 Barcelona, Spain.

[6] Department of Animal Science, AGRIS Sardegna, Loc. Bonassai, 07100 Sassari, Italy

[7] Department of Agriculture, University of Sassari, Viale Italia, 39A, 07100 Sassari, Italy

***Correspondence:** sanna@uniss.it; Tel.: (+39) 079229500.

Supplementary Material

Table S1. Operational conditions of microwave acid digestion and dry ashing for honey elemental analysis. 3

Table S2. Inductively Coupled Plasma – Mass Spectrometry (ICP-MS) instrumental conditions for honey elemental analysis. 4

Table S3. Elemental settings and validation parameters for the ICP-MS determination of macroelements and lanthanides in honey. 5

Table S4. Trueness evaluation for the ICP-MS determination of macroelements and lanthanides by means analysis of certified reference materials (NIST SRM 1515 apple leaves and BCR 668 mussel tissue). 6

Table S5. Concentration of macroelements, trace elements, toxic elements and lanthanides in honeys from Sardinia and Spain (min; mean ± sd; max). 7

Table S6. Random forest (RF) accuracy table for each set of predictors tested: botanical origin. 9

Table S7. RF accuracy table for each set of predictors tested: geographical and botanical origin. 10

Table S8. RF accuracy table for each set of predictors tested: geographical origin. 11

Figure S1. Principal Component Analysis (PCA) performed using autoscaling as data pre-treatment. (A) Loading plot; (B) Score plot. 12

Figure S2. Proportion of variance (PCA using centered log-ratio transformation). 13

Table S1. Operational conditions of microwave acid digestion and dry ashing for honey elemental analysis.

Microwave acid digestion (ultraWAVE Milestone)

Step		Time (min)	Temperature (°C)
1st	Heating	25	240
2nd	Holding	10	240
3rd	Cooling	ca. 30	< 40

Dry ashing (Controller P320 Nabertherm)

Step		Time (min)	Temperature (°C)
1st	Heating	10	105
2nd	Heating	30	150
3rd	Holding	30	150
4th	Heating	30	200
5th	Holding	30	200
6th	Heating	360	600
7th	Holding	360	600

Table S2. Inductively Coupled Plasma – Mass Spectrometry (ICP-MS) instrumental conditions for honey elemental analysis.

ICP-MS NexION 300X Perkin Elmer settings			
RF power	1300	KED mode cell entrance	-8.0
Ar plasma flow	18.0	KED mode cell exit	-25.0
Ar auxiliary flow	1.20	Resolution (Da)	0.7
Ar nebulizer flow	0.91	Scan mode	Peak hopping
Nebulizer	Meinhardt®, glass	Detector mode	Dual
Spray chamber	Cyclonic, glass	Dwell time (ms)	50
Skimmer and	Nickel	Number of points per	3
Sampling depth	0	Acquisition time (s)	6
Deflector voltage	-8.00	Acquisition dead time	35
Analog stage	-1750	KED gas	Helium 99.999%
Pulse stage voltage	1350	Masses of optimization	^{7}Li, ^{115}In and ^{205}Tl

Table S3. Elemental settings and validation parameters for the ICP-MS determination of macroelements and lanthanides in honey.

Macroelements

Element	Mode	Calibration Range (mg dm^{-3})	R^2	LoD (mg kg^{-1})	LoQ (mg kg^{-1})	CV%$_r$	CV%$_{IP}$
^{23}Na	KED	0.05 - 5	0.9998	0.10	0.30	6	8
^{24}Mg	KED	0.01 – 10	0.9996	0.26	0.80	2	5
^{39}K	KED	0.1 – 100	0.9998	0.70	0.20	2	6
^{43}Ca	KED	0.05 - 50	0.9997	0.16	0.50	2	5

He flow rate = 3.6 cm^3 min^{-1}

Lanthanides

Element	Mode	Calibration Range (μg dm^{-3})	R^2	LoD (μg kg^{-1})	LoQ (μg kg^{-1})	CV%$_r$	CV%$_{IP}$
^{139}La	KED	0.001 - 10	0.9999	0.015	0.050	15	21
^{140}Ce	KED	0.001 - 10	0.9998	0.024	0.079	15	18
^{141}Pr	KED	0.001 - 10	0.9999	0.006	0.020	15	18
^{142}Nd	KED	0.001 - 10	0.9993	0.018	0.059	16	19
^{152}Sm	KED	0.001 - 10	0.9997	0.003	0.010	11	16
^{153}Eu	KED	0.001 - 10	0.9998	0.003	0.010	15	16
^{158}Gd	KED	0.001 - 10	0.9999	0.003	0.010	6	15
^{159}Tb	KED	0.001 - 10	0.9996	0.001	0.003	7	13
^{164}Dy	KED	0.001 - 10	0.9999	0.003	0.010	8	12
^{165}Ho	KED	0.001 - 10	0.9999	0.003	0.010	12	16
^{166}Er	KED	0.001 - 10	0.9996	0.003	0.010	8	18
^{169}Tm	KED	0.001 - 10	0.9999	0.001	0.003	3	16
^{174}Yb	KED	0.001 - 10	0.9999	0.001	0.004	10	17
^{175}Lu	KED	0.001 - 10	0.9998	0.001	0.002	5	18

He flow rate = 4.6 cm^3 min^{-1}

Macroelements and lanthanides were determined after microwave acid digestion and dry ashing, respectively.

Detailed information about the instrumental parameters and elemental settings for trace and toxic elements analysis, method assessment, performance, quality control, and validation can be found in ref. [38].

Table S4. Trueness evaluation for the ICP-MS determination of macroelements and lanthanides by means of analysis of certified reference materials (NIST SRM 1515 apple leaves and BCR 668 mussel tissue).

NIST SRM 1515 apple leaves

Element	Certified value (mg kg^{-1})	Experimental value (mg kg^{-1}) (n=3)	Recovery %*
Na	24.4 ± 2.1	23 ± 3	105 ± 15
Mg	2710 ± 120	2600 ± 200	104 ± 7
K	16080 ± 210	15300 ± 900	105 ± 6
Ca	15250 ± 100	15700 ± 900	97 ± 6

BCR 668 mussel tissue

Element	Certified value (μg kg^{-1})	Experimental value (μg kg^{-1}) (n=3)	Recovery %*
La	80 ± 6	75 ± 4	94 ± 8
Ce	89 ± 7	84 ± 9	90 ± 10
Pr	12.3 ± 1.1	11.9 ± 1.5	100 ± 10
Nd	54 ± 4	54 ± 9	100 ± 30
Sm	11.2 ± 0.8	11.9 ± 0.9	110 ± 10
Eu	2.79 ± 0.16	2.6 ± 0.5	90 ± 20
Gd	13.0 ± 0.6	14 ± 1	110 ± 10
Tb	1.62 ± 0.12	1.73 ± 0.08	107 ± 9
Dy	8.9 ± 0.6	9.7 ± 0.3	109 ± 8
Ho	1.8 ± 0.6	1.9 ± 0.8	105 ± 20
Er	4.5 ± 0.5	4.3 ± 0.2	100 ± 10
Tm	0.48 ± 0.08	0.5 ± 0.03	110± 20
Yb	2.8 ± 0.5	2.8 ± 0.2	100 ± 20
Lu	0.389 ± 0.024	0.44 ± 0.04	110 ± 10

* Recoveries has been calculated using unrounded experimental concentrations

Table S5. Concentration of macroelements, trace elements, toxic elements and lanthanides in honeys from Sardinia and Spain (min; mean ± sd; max).

Macroelements(mg kg^{-1})

Elements	SPA ROS (n = 26)	SPA MUL (n = 34)	SPA EUC (n = 13)	ITA THI (n = 39)	ITA STR (n = 31)	ITA ROS (n = 6)	ITA MUL (n = 35)	ITA EUC (n = 30)	ITA ASP (n = 33)
Na	3; 20 ± 10; 35	4; 20 ± 10; 48	13; 40 ± 10; 56	11; 50 ± 20; 99	3; 20 ± 20; 68	6.1; 8 ± 3; 12.9	10; 60 ± 40; 150	20; 110 ± 60; 240	1; 16 ± 8; 37
Mg	2; 12 ± 6; 29	3; 30 ± 20; 85	7; 40 ± 15; 62	1; 9 ± 4; 19	2; 12 ± 8; 32	3.5; 5 ± 1.5; 6.6	4; 12 ± 7; 27	2; 13 ± 5; 23	2; 7 ± 4; 16
K	60; 300 ± 150; 650	< LoD; 600 ± 400; 1700	200; 1000 ± 400; 1600	90; 600 ± 300; 1270	200; 1500 ± 800; 3000	110; 190 ± 90; 330	140; 550 ± 300; 1080	70; 500 ± 200; 880	10; 200 ± 90; 400
Ca	4; 30 ± 10; 51	8; 50 ± 25; 97	11; 80 ± 30; 120	4; 30 ± 10; 59	10; 70 ± 40; 150	11; 20 ± 20; 52	10; 35 ± 15; 70	10; 70 ± 30; 130	2; 14 ± 8; 33

Lanthanides (ng kg^{-1})

Elements	SPA ROS (n = 26)	SPA MUL (n = 34)	SPA EUC (n = 13)	ITA THI (n = 39)	ITA STR (n = 31)	ITA ROS (n = 6)	ITA MUL (n = 35)	ITA EUC (n = 30)	ITA ASP (n = 33)
La	200; 1100 ± 300; 1800	800; 1600 ± 800; 3500	900; 2100 ± 900; 4100	1200; 5000 ± 2000; 9400	800; 300 ± 2000; 7900	360; 360 ± 10; 370	400; 1300 ± 800; 3300	1000; 7000 ± 3000; 14000	200; 1100 ± 500; 2000
Ce	300; 1400 ± 400; 2300	900; 1600 ± 500; 3100	1300; 3000 ± 1500; 6100	2000; 8000 ± 4000; 18000	2000; 5000 ± 3000; 15000	400; 800 ± 200; 900	400; 2000 ± 1000; 4600	1000; 12000 ± 6000; 23000	300; 1400 ± 600; 2700
Pr	100; 300 ± 100; 400	200; 400 ± 200; 1000	200; 600 ± 300; 1200	300; 1300 ± 600; 2600	200; 900 ± 600; 2500	94; 95 ± 1; 97	100; 300 ± 200; 800	200; 1900 ± 900; 3900	100; 300 ± 100; 600
Nd	400; 1000 ± 300; 1700	600; 1500 ± 900; 3700	900; 2500 ± 1000; 5100	1300; 5500 ± 3000; 11400	900; 4000 ± 3000; 10800	300; 500 ± 200; 700	300; 1400 ± 800; 3100	1000; 8000 ± 4000; 15000	300; 1100 ± 500; 2200
Sm	100; 200 ± 100; 300	100; 300 ± 200; 1000	200; 600 ± 300; 1200	300; 1100 ± 600; 2400	200; 800 ± 600; 2300	100; 100 ± 100; 200	100; 400 ± 300; 1200	100; 1600 ± 700; 3000	100; 200 ± 100; 500
Eu	20; 100 ± 30; 140	100; 300 ± 200; 600	100; 300 ± 300; 800	100; 600 ± 300; 1200	100; 400 ± 300; 1000	100; 100 ± 100; 200	100; 500 ± 400; 1500	100; 700 ± 300; 1100	< LoD; 100 ± 100; 200
Gd	< LoD; 200 ± 10; 210	100; 200 ± 100; 700	100; 500 ± 200; 900	200; 900 ± 400; 1800	200; 700 ± 500; 1800	100; 100 ± 100; 200	100; 400 ± 200; 800	100; 1300 ± 600; 2600	< LoD; 200 ± 100; 400
Tb	10; 20 ± 10; 21	20; 40 ± 20; 90	< LoD; 100 ± 2; 110	< LoD; 100 ± 100; 200	< LoD; 100 ± 100; 200	23.6; 23.9 ± 0.3; 24.2	< LoD; 100 ± 100; 200	< LoD; 200 ± 100; 300	10; 30 ± 10; 50
Dy	30; 110 ± 30; 150	100; 200 ± 100; 500	100; 300 ± 200; 600	100; 600 ± 300; 1200	100; 400 ± 300; 1100	50; 90 ± 30; 130	< LoD; 300 ± 100; 600	< LoD; 800 ± 300; 1500	< LoD; 100 ± 100; 200
Ho	10; 20 ± 10; 30	10; 30 ± 20; 80	20; 50 ± 30; 100	< LoD; 100 ± 100; 200	< LoD; 100 ± 100; 200	19.2; 19.5 ± 0.2; 19.8	20; 40 ± 20; 90	< LoD; 100 ± 100; 200	10; 20 ± 10; 30
Er	10; 50 ± 10; 70	< LoD; 100 ± 100; 200	< LoD; 100 ± 100; 200	100; 200 ± 100; 500	< LoD; 200 ± 100; 400	29; 29 ± 1; 29	< LoD; 100 ± 100; 200	< LoD; 300 ± 100; 600	10; 50 ± 20; 100
Tm	6; 7 ± 10; 9	< LoD; 10 ± 10; 30	10; 20 ± 10; 30	10; 30 ± 20; 6 0	10; 20 ± 20; 60	3.6; 3.6 ± 0.1; 3.7	< LoD; 10 ± 1; 11	10; 50 ± 20; 80	4; 7 ± 3; 14
Yb	10; 40 ± 10; 60	< LoD; 100 ± 10; 200	< LoD; 100 ± 100; 200	< LoD; 200 ± 100; 400	< LoD; 100 ± 100; 400	24.6; 24.9 ± 0.3; 25.2	< LoD; 100 ± 100; 200	< LoD; 300 ± 100; 500	10; 40 ± 20; 70
Lu	5; 5 ± 1; 7	< LoD; 10 ± 10; 20	< LoD; 10 ± 10; 30	10; 30 ± 20; 60	10; 20 ± 20; 60	4.3; 4.4 ± 0.1; 4.4	< LoD; 10 ± 10; 20	< LoD; 40 ± 20; 80	3; 7 ± 3; 12

Trace and toxic elements (μg kg^{-1})

Elements	SPA ROS (n = 26)	SPA MUL (n = 34)	SPA EUC (n = 13)	ITA THI (n = 39)	ITA STR (n = 31)	ITA ROS (n = 6)	ITA MUL (n = 35)	ITA EUC (n = 30)	ITA ASP (n = 33)
Co	1.2; 3 ± 2; 7	2; 11 ± 9; 34	6; 14 ± 6; 24	1.4; 5 ± 3; 13.2	1.1; 1.8 ± 0.1; 3	4; 6 ± 3; 9.1	1.1; 3 ± 1; 7	2; 4 ± 1; 6.7	1.2; 3 ± 1; 6.4
Cr	< LoD	< LoD	< LoD	< LoD	< LoD	< LoD	< LoD	< LoD	< LoD
Cu	170; 220 ± 80; 490	100; 400 ± 300; 1400	100; 700 ± 400; 1400	70; 130 ± 10; 240	80; 110 ± 10; 190	110; 150 ± 10; 200	90; 180 ± 70; 340	80; 140 ± 10; 220	70; 120 ± 10; 180
Fe	< LoD; 1500 ± 300; 2100	< LoD; 2400 ± 1000; 3600	< LoD; 2800 ± 900; 4400	120; 300 ± 200; 730	110; 190 ± 10; 360	< LoD	590; 800 ± 300; 1420	100; 500 ± 200; 840	100; 180 ± 10; 320
Mn	50; 300 ± 200; 710	< LoD; 3000 ± 3000; 12000	900; 4000 ± 2000; 7200	40; 200 ± 100; 460	40; 200 ± 100; 470	40; 110 ± 80; 240	70; 300 ± 100; 730	100; 3000 ± 1500; 5100	40; 120 ± 70; 330
Mo	< LoD	< LoD	4.6; 5.4 ± 0.1; 6.6	< LoD	< LoD	< LoD	< LoD	< LoD	< LoD
Ni	18; 30 ± 10; 46	20; 70 ± 60; 280	20; 200 ± 100; 480	11; 22 ± 1; 44	10; 16 ± 1; 28	16; 25 ± 10; 40	11; 20 ± 10; 52	11; 18 ± 1; 34	13; 22 ± 1; 34
Sr	50; 120 ± 60; 270	60; 150 ± 60; 270	70; 210 ± 60; 270	18; 80 ± 20; 128	20; 130 ± 50; 240	37; 60 ± 20; 77	20; 80 ± 30; 170	20; 180 ± 60; 290	10; 30 ± 10; 56
V	3; 12 ± 5; 18	< LoD	2.3; 7 ± 3; 10.6	0.8; 1.2 ± 0.1; 1.9	0.7; 0.8 ± 0.1; 1	< LoD	1.1; 1.6 ± 0.1; 2.6	1; 4 ± 1; 7.3	< LoD
Zn	560; 900 ± 200; 1370	400; 1100 ± 400; 2100	700; 1800 ± 700; 2800	300; 700 ± 300; 1480	260; 600 ± 200; 1180	420; 600 ± 200; 850	400; 800 ± 400; 1900	330; 600 ± 150; 1000	250; 500 ± 200; 1000
Ag	< LoD	< LoD	< LoD	< LoD	< LoD	< LoD	< LoD	< LoD	< LoD
Ba	118; 138 ± 1; 151	100; 200 ± 100; 500	170; 500 ± 200; 720	80; 200 ± 100; 520	100; 500 ± 400; 1300	< LoD	110; 150 ± 10; 270	80; 300 ± 100; 540	70; 190 ± 90; 340
Li	< LoD	< LoD	< LoD	< LoD	7.3; 12.1 ± 0.1; 16.7	< LoD	< LoD	7; 12 ± 1; 20	< LoD
As	< LoD	< LoD	8; 9 ± 1; 11	< LoD	< LoD	< LoD	< LoD	7.5; 8.8 ± 0.1; 10.3	< LoD
Be	< LoD	< LoD	< LoD	< LoD	< LoD	< LoD	< LoD	< LoD	< LoD
Bi	< LoD	< LoD	< LoD	< LoD	< LoD	< LoD	< LoD	< LoD	< LoD
Cd	< LoD	1.3; 1.9 ± 0.1; 3.1	1; 1.9 ± 0.1; 2.8	1.1; 1.6 ± 0.1; 2.6	< LoD	< LoD	1.2; 1.9 ± 0.1; 3.5	1; 1.9 ± 0.1; 3.4	< LoD
Hg	< LoD	< LoD	< LoD	< LoD	< LoD	< LoD	< LoD	< LoD	< LoD
Pb	< LoD	< LoD	< LoD	10.2; 12.7 ± 0.1; 15.1	< LoD	< LoD	< LoD	14; 19 ± 1; 27	13.1; 13.4 ± 0.1; 13.6
Sb	< LoD	< LoD	< LoD	< LoD	< LoD	< LoD	< LoD	< LoD	< LoD
Sn	9.1; 12.8 ± 0.1; 21	7; 15 ± 1; 29	10; 14 ± 1; 19	10; 40 ± 30; 120	10; 40 ± 40; 140	19; 24 ± 1; 27	8; 17 ± 8; 29	7; 30 ± 20; 88	11; 40 ± 30; 104
Te	< LoD	< LoD	< LoD	< LoD	< LoD	< LoD	< LoD	< LoD	< LoD
Tl	< LoD	1.6; 4 ± 2; 7.1	1; 1.7 ± 0.8; 3.1	< LoD; 0.20 ± 0.01; 0.21	0.13; 0.1 ± 0.1; 0.15	< LoD	< LoD	< LoD	< LoD; 0.20 ± 0.01; 0.21

SPA, Spain, ITA, Sardinia, Italy; ROS, rosemary; MUL, multifloral; EUC, eucalyptus; THI, thistle; STR, strawberry tree; ASP, asphodel.

Table S6. Random forest (RF) accuracy table for each set of predictors tested: botanical origin.

		ASP		EUC		MUL		ROS		STR		THI	
Index	Predictors	Training	Test	Training	Test	Training	Test	Training	Test	Training	Test	Training	Test
21	Na, Mg, K, Ca, Mn, Sr, Zn, La, Ce, Pr, Nd, Sm, Eu, Gd, Tb, Dy, Ho, Er, Tm, Yb, Lu	73±9	70±10	78±10	80±9	40±10	47±7	70±10	70±10	70±10	70±10	74±9	80±10
20	Na, Mg, K, Ca, Mn, Sr, Zn, La, Ce, Pr, Nd, Eu, Gd, Tb, Dy, Ho, Er, Tm, Yb, Lu	72±10	75±10	77±10	79±8	45±10	47±9	70±10	70±10	70±10	70±10	77±9	70±10
19	Na, Mg, K, Ca, Mn, Sr, Zn, La, Ce, Pr, Nd, Eu, Gd, Tb, Dy, Er, Tm, Yb, Lu	73±10	80±10	80±10	79±10	40±10	48±8	70±10	75±10	70±10	70±10	74±8	75±9
18	Na, Mg, K, Ca, Mn, Sr, Zn, La, Ce, Pr, Nd, Eu, Gd, Tb, Dy, Tm, Yb, Lu	73±10	75±10	79±9	80±8	50±10	46±8	70±10	80±10	70±10	70±10	76±8	80±10
17	Na, Mg, K, Ca, Mn, Sr, Zn, La, Ce, Pr, Nd, Eu, Gd, Tb, Dy, Tm, Lu	74±10	80±10	79±8	82±8	50±10	47±8	80±10	75±10	71±9	70±10	76±8	77±9
16	Na, Mg, K, Ca, Mn, Sr, Zn, Ce, Pr, Nd, Eu, Gd, Tb, Dy, Tm, Lu	74±9	80±10	80±10	79±9	40±10	47±8	70±10	80±10	71±9	70±10	75±8	80±10
15	Na, Mg, K, Ca, Mn, Sr, Zn, Ce, Pr, Nd, Eu, Tb, Dy, Tm, Lu	75±10	75±10	80±9	80±8	50±10	46±8	73±9	80±10	70±10	70±10	76±9	80±10
14	Na, K, Ca, Mn, Sr, Zn, Ce, Pr, Nd, Eu, Tb, Dy, Tm, Lu	72±8	80±10	79±9	80±10	50±10	49±7	74±9	80±10	70±10	70±10	75±8	80±10
13	Na, K, Ca, Mn, Sr, Zn, Ce, Pr, Nd, Eu, Tb, Dy, Lu	75±10	78±9	80±10	81±9	50±10	49±7	70±10	80±10	70±10	70±10	75±9	80±10
12	Na, K, Ca, Mn, Sr, Zn, Ce, Nd, Eu, Tb, Dy, Lu	77±9	80±10	80±10	80±9	50±10	49±8	73±9	80±10	74±9	70±10	76±8	75±10
11	Na, K, Ca, Mn, Sr, Zn, Ce, Nd, Eu, Tb, Lu	76±9	80±10	80±9	81±9	50±10	48±8	72±8	75±10	73±9	80±10	77±9	80±10
10	Na, K, Ca, Mn, Sr, Ce, Nd, Eu, Tb, Lu	75±10	78±9	80±9	81±8	45±10	47±9	72±9	70±10	74±9	80±10	77±8	80±10
9	Na, K, Ca, Mn, Sr, Ce, Eu, Tb, Lu	74±10	80±10	80±10	80±9	45±10	47±8	70±10	75±10	70±10	80±10	76±9	80±10
8	Na, K, Ca, Mn, Sr, Ce, Eu, Lu	77±9	79±9	80±10	81±9	45±10	47±7	70±10	70±10	78±8	80±10	76±9	78±9
7	K, Ca, Mn, Sr, Ce, Eu, Lu	77±9	80±10	80±8	82±9	40±10	47±8	70±10	70±10	69±9	70±10	75±9	77±9
6	K, Mn, Sr, Ce, Eu, Lu	74±8	74±10	78±9	77±9	50±10	50±8	70±10	70±10	70±10	70±10	74±9	75±10
5	K, Mn, Sr, Ce, Lu	71±9	70±10	76±9	77±8	40±10	37±8	65±10	60±10	70±10	70±10	72±9	70±10
4	K, Mn, Sr, Lu	71±9	73±10	80±10	80±10	35±10	39±9	60±10	65±10	69±9	70±10	71±9	70±10
3	Mn, Sr, Lu	72±10	71±10	78±9	78±8	30±10	38±8	60±10	60±10	60±10	50±10	60±10	60±10
2	Mn, Lu	58±10	60±10	80±10	76±10	40±10	40±9	60±10	60±10	40±10	40±10	50±10	55±10

Values in tables S6-S8 represent the percentage of accuracy in classifying the data in the test and the training datasets. The values have been rounded according to the relevant standard deviation.

Table S7. RF accuracy table for each set of predictors tested: geographical and botanical origin.

Inde x	Predictors	ITA ASP		ITA EUC		ITA MUL		ITA ROS		ITA STR		ITA THI		SPA EUC		SPA MUL		SPA ROS	
		Training	Test	Training	Test	Training	Test	Training	Test	Training	Test	Training	Test	Training	Test	Training	Test	Training	Test
21	Na, Mg, K, Ca, Mn, Sr, Zn, La, Ce, Pr, Nd, Sm, Eu, Gd, Tb, Dy, Ho, Er, Tm, Yb, Lu	80±10	80±10	82±8	83±9	60±10	65±10	20±30	40±20	70±10	70±10	70±10	70±10	45±25	50±20	50±10	50±10	73±8	80±10
20	Na, Mg, K, Ca, Mn, Sr, Zn, La, Ce, Pr, Nd, Sm, Eu, Gd, Tb, Dy, Er, Tm, Yb, Lu	70±10	75±10	81±9	80±10	66±9	60±10	30±35	35±20	70±10	70±10	70±9	72±9	40±30	50±15	50±10	50±10	74±9	80±10
19	Na, Mg, K, Ca, Mn, Sr, Zn, La, Ce, Pr, Nd, Eu, Gd, Tb, Dy, Er, Tm, Yb, Lu	75±10	75±101	81±9	80±10	60±10	70±10	30±30	40±20	70±10	70±10	70±10	70±10	50±30	50±20	50±10	50±10	75±10	80±10
18	Na, Mg, K, Ca, Mn, Sr, Zn, La, Ce, Pr, Nd, Eu, Gd, Tb, Dy, Tm, Yb, Lu	75±9	78±9	83±7	80±10	65±10	70±10	30±33	40±20	70±10	70±10	71±9	72±9	50±25	50±15	50±10	50±10	75±8	80±10
17	Na, Mg, K, Ca, Mn, Sr, Zn, La, Ce, Pr, Nd, Eu, Tb, Dy, Tm, Yb, Lu	75±9	80±10	82±8	82±9	65±10	70±10	20±30	40±20	70±10	70±10	70±10	70±10	40±25	50±20	50±10	55±10	75±8	80±10
16	Na, Mg, K, Ca, Mn, Sr, Zn, La, Ce, Pr, Nd, Eu, Tb, Dy, Tm, Lu	77±9	80±10	82±8	84±8	60±10	70±10	30±30	40±20	70±10	70±10	72±8	70±10	50±20	50±10	50±10	55±10	75±10	80±10
15	Na, Mg, K, Ca, Mn, Sr, Zn, La, Ce, Pr, Nd, Eu, Tb, Tm, Lu	78±9	77±9	82±8	83±9	65±10	70±10	20±30	40±20	70±10	70±10	70±10	70±10	50±20	50±15	50±10	50±10	77±9	80±10
14	Na, Mg, K, Ca, Mn, Sr, Zn, La, Ce, Pr, Nd, Eu, Tb, Lu	80±10	78±9	83±9	80±10	70±10	70±10	30±30	40±20	70±10	75±10	70±9	74±9	50±20	50±15	50±15	55±10	76±9	80±10
13	Na, K, Ca, Mn, Sr, Zn, La, Ce, Pr, Nd, Eu, Tb, Lu	80±10	80±10	83±8	80±10	70±10	70±10	30±35	40±20	70±10	70±10	71±9	70±10	40±20	60±20	50±10	50±10	78±8	80±10
12	Na, K, Ca, Mn, Sr, Zn, Ce,Pr, Nd, Eu, Tb, Lu	78±9	80±10	84±8	80±10	70±10	70±10	30±30	40±20	70±10	70±10	73±9	70±10	50±20	50±20	50±10	60±10	78±9	80±10
11	Na, K, Ca, Mn, Sr, Zn, Ce, Nd, Eu, Tb, Lu	78±9	79±8	83±8	84±8	70±10	70±10	20±30	30±20	70±10	70±10	70±10	70±10	45±20	50±20	50±10	50±10	77±9	80±10
10	Na, K, Ca, Mn, Sr, Ce, Nd, Eu, Tb, Lu	80±10	79±8	83±8	84±9	70±10	70±10	15±30	40±20	70±10	75±10	74±9	73±9	40±20	50±20	50±10	60±10	76±9	80±10
9	Na, K, Ca, Mn, Sr, Ce, Nd, Eu, Lu	77±8	80±10	84±7	80±10	60±10	66±9	20±30	30±20	70±10	70±10	72±9	70±10	50±20	60±10	50±10	50±10	73±9	80±10
8	Na, K, Mn, Sr, Ce, Nd, Eu, Lu	75±10	77±9	83±8	82±9	65±10	70±10	20±30	30±20	70±10	70±10	70±10	72±9	40±20	50±15	50±10	50±10	75±10	80±10
7	Na, K, Mn, Sr, Ce, Eu, Lu	75±10	78±9	83±7	84±9	65±10	69±9	20±30	30±20	70±10	70±10	69±8	70±10	40±30	50±20	50±10	50±10	74±9	80±10
6	Na, K, Mn, Sr, Ce, Lu	70±10	70±10	80±10	80±10	60±10	60±10	20±30	35±30	70±10	80±10	70±10	70±10	50±20	60±15	50±10	50±10	70±10	70±10
5	Na, Mn, Sr, Ce, Lu	71±9	70±10	80±7	82±9	60±10	60±10	20±30	40±25	60±10	60±10	60±10	60±10	50±25	60±20	50±10	50±10	65±10	70±15
4	Mn, Sr, Ce, Lu	70±10	70±10	78±9	80±10	50±10	50±10	10±20	30±25	50±10	50±10	60±10	60±10	40±20	50±20	50±10	55±10	60±10	60±10
3	Mn, Sr, Lu	70±10	70±10	80±10	80±10	50±10	60±10	10±20	20±20	55±10	50±10	60±10	60±10	40±20	45±15	50±10	50±10	60±10	60±10
2	Mn, Lu	55±10	60±10	80±10	80±10	60±10	60±10	20±30	30±20	40±10	40±10	50±10	50±10	30±20	40±20	50±10	50±10	60±10	60±10

Table S8. RF accuracy table for each set of predictors tested: geographical origin.

		ITA		SPA	
**Index**	**Predictors**	**Training**	**Test**	**Training**	**Test**
21	Na, Mg, K, Ca, Mn, Sr, Zn, La, Ce, Pr, Nd, Sm, Eu, Gd, Tb, Dy, Ho, Er, Tm, Yb, Lu	89±5	88±5	90±3	89±6
20	Na, Mg, K, Ca, Mn, Sr, Zn, La, Ce, Pr, Nd, Sm, Eu, Gd, Tb, Dy, Er, Tm, Yb, Lu	89±4	90±3	88±3	89±7
19	Na, Mg, Ca, Mn, Sr, Zn, La, Ce, Pr, Nd, Sm, Eu, Gd, Tb, Dy, Er, Tm, Yb, Lu	88±4	88±4	89±4	85±8
18	Na, Mg, Ca, Mn, Sr, Zn, La, Ce, Pr, Nd, Sm, Eu, Gd, Tb, Dy, Er, Tm, Lu	88±5	88±4	90±3	89±5
17	Na, Mg, Ca, Mn, Sr, Zn, La, Ce, Pr, Nd, Sm, Eu, Gd, Tb, Dy, Tm, Lu	87±4	89±3	88±3	86±8
16	Na, Mg, Ca, Mn, Sr, Zn, La, Ce, Pr, Nd, Sm, Eu, Gd, Tb, Tm, Lu	89±2	91±4	89±4	86±8
15	Na, Mg, Ca, Mn, Sr, Zn, La, Ce, Pr, Nd, Sm, Eu, Gd, Tb, Lu	88±5	91±3	89±4	91±5
14	Na, Mg, Ca, Mn, Sr, Zn, Ce, Pr, Nd, Sm, Eu, Gd, Tb, Lu	88±5	90±3	88±4	90±10
13	Na, Mg, Ca, Mn, Sr, Zn, Ce, Pr, Nd, Eu, Gd, Tb, Lu	89±5	88±5	87±5	91±5
12	Na, Mg, Mn, Sr, Zn, Ce, Pr, Nd, Eu, Gd, Tb, Lu	89±5	88±3	87±4	94±5
11	Na, Mg, Mn, Sr, Zn, Ce, Nd, Eu, Gd, Tb, Lu	89±4	89±5	89±5	89±5
10	Na, Mg, Mn, Sr, Zn, Ce, Nd, Eu, Gd, Tb	88±3	89±5	89±5	91±4
9	Na, Mg, Mn, Sr, Zn, Ce, Nd, Eu, Tb	91±3	88±4	92±3	91±5
8	Na, Mg, Mn, Sr, Zn, Ce, Nd, Tb	86±5	90±3	89±5	89±9
7	Mg, Mn, Sr, Zn, Ce, Nd, Tb	88±5	90±3	88±4	90±5
6	Mn, Sr, Zn, Ce, Nd, Tb	86±5	88±5	86±3	90±5
5	Mn, Zn, Ce, Nd, Tb	84±4	84±4	89±3	89±5
4	Mn, Zn, Ce, Tb	86±6	84±4	85±3	88±6
3	Mn, Zn, Ce	86±4	89±3	88±4	81±6
2	Mn, Zn	77±8	73±7	76±7	80±10

Figure S1. Principal Component Analysis (PCA) performed using autoscaling as data pre-treatment. (A) Loading plot; (B) Score plot.

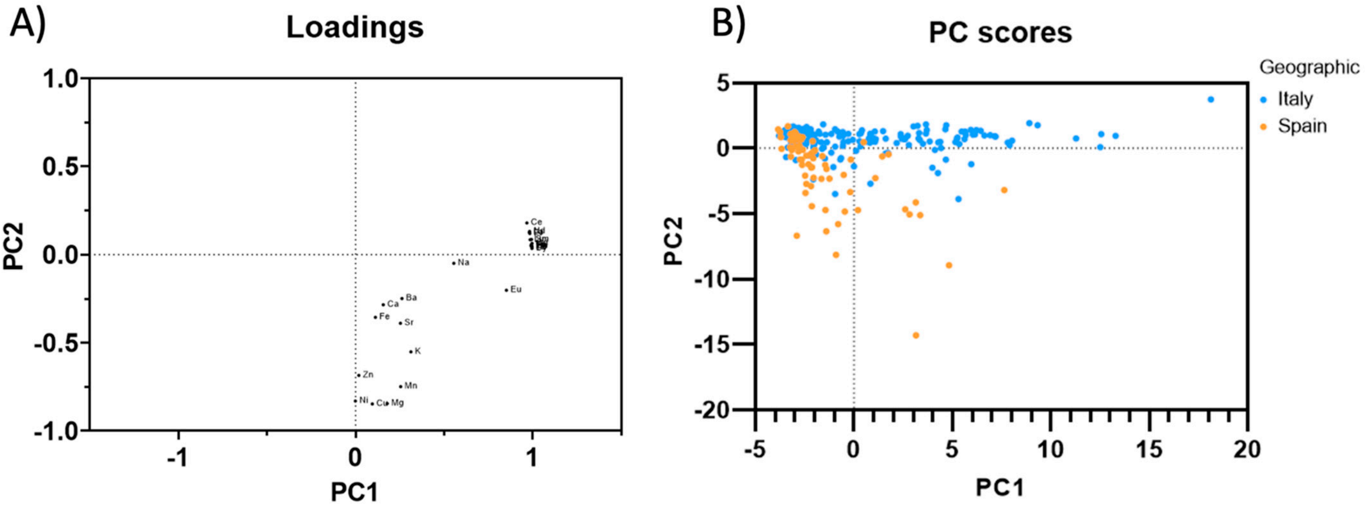

Figure S2. Proportion of variance (PCA using centered log-ratio transformation).

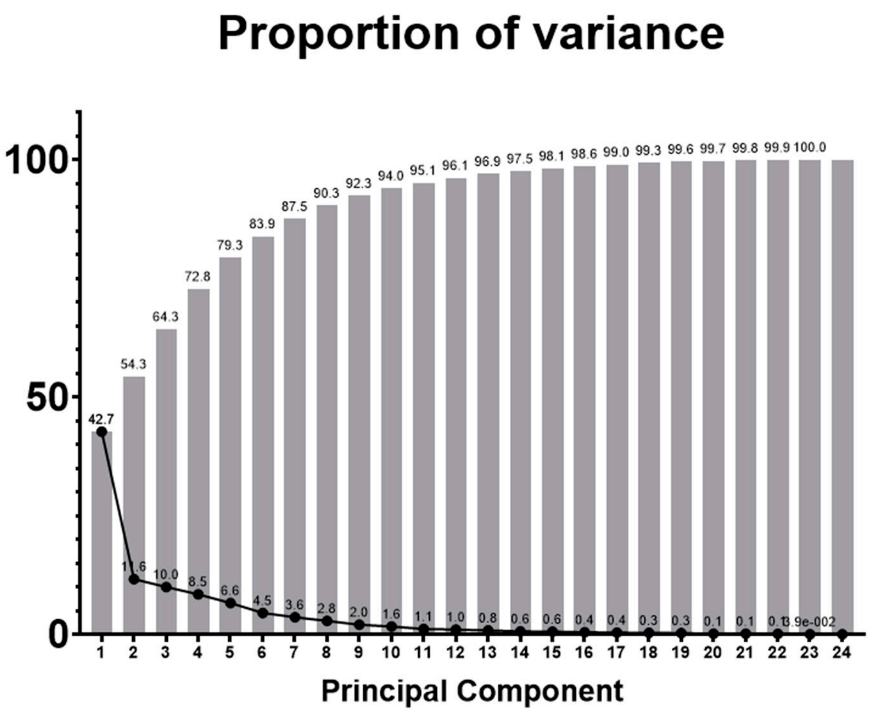

Supplement: Supplementary file 1 [file foods-13-00243-s001.zip › foods-2799774-supplementary.pdf]
